# Dual roles and therapeutic potential of Keap1-Nrf2 pathway in pancreatic cancer: a systematic review

**DOI:** 10.1186/s12964-019-0435-2

**Published:** 2019-09-11

**Authors:** Jiang-Jiang Qin, Xiang-Dong Cheng, Jia Zhang, Wei-Dong Zhang

**Affiliations:** 1 College of Pharmaceutical Science, Zhejiang Chinese Medical University, 548 Binwen Road, Binjiang District, Hangzhou, 310053 Zhejiang China; 2 Zhejiang Cancer Hospital, Hangzhou, 310022 China; 3 Shanxi Institute of Traditional Chinese Medicine, Taiyuan, 030012 China; 4 School of Pharmacy, Naval Medical University, 325 Guohe Road, Yangpu District, Shanghai, 200433 China; 5 Institute of Interdisciplinary Integrative Medicine Research, Shanghai University of Traditional Chinese Medicine, Shanghai, 201203 China

**Keywords:** Keap1, Nrf2, Pancreatic cancer, Tumor-suppressive and promoting roles, Small molecule activators and inhibitors, Prevention and therapy

## Abstract

Pancreatic cancer (PC) is one of the most fatal diseases with a very high rate of metastasis and low rate of survival. Despite the advances in understanding this devastating disease, PC still accounts for 3% of all cancers and causes almost 7% of death of cancer patients. Recent studies have demonstrated that the transcription factor nuclear factor-erythroid 2-related factor 2 (Nrf2) and its key negative regulator Kelch-like ECH-associated protein 1 (Keap1) are dysregulated in PC and the Keap1-Nrf2 pathway is an emerging target for PC prevention and therapy. Indeed, Nrf2 plays an either tumor-suppressive or promoting function in PC, which depends on the developmental stages of the disease and the cellular context. Several natural-product Nrf2 activators have been developed to prevent pancreatic carcinogenesis, while the Nrf2 inhibitors have been examined for their efficacy in inhibiting PC growth and metastasis and reversing chemoresistance. However, further preclinical and clinical studies for determining the effectiveness and safety of targeting the Keap1-Nrf2 pathway for PC prevention and therapy are warranted. In this review, we comprehensively discuss the dual roles of the Keap1-Nrf2 signaling pathway in PC as well as the current targeting strategies and known activators and inhibitors of Nrf2. We also propose new strategies that may be used to address the current issues and develop more specific and more effective Nrf2 activator/inhibitors for PC prevention and therapy.

## Background

Pancreatic cancer (PC) remains the most aggressive and most lethal cancer with the worst survival and prognosis, although significant advances have been made in PC research, especially in its molecular mechanisms, epidemiology, pathophysiology, and treatment in the past decades [1–4]. Because the initiation of PC is often quite nonspecific and subtle, this notorious disease is very difficult to be diagnosed in its early phases and it is always detected at the advanced stages with metastasis [3]. Since only about 10 to 15% PC are diagnosed at the resectable stage and can be treated with surgery, many patients prolong their lives by relying on radiotherapy and chemotherapy, which may also decrease the quality of their lives due to the side effects [5]. Numerous studies on molecular abnormalities in PC have been performed, leading to the identification of several driver mutations, such as *KRAS*, *TP53*, *CDKN2A*, and *SMAD4*, which contribute to the initiation and development of PC [6, 7]. Targeted agents that specifically inhibit these molecular changes have been developed, e.g. Erlotinib and Larotrectinib [8]. Although these targeted therapies alone or in combination with chemotherapy, e.g. gemcitabine have shown some efficacy in patients with unresectable PC, only a very limited increase in survival time of patients has been observed [8]. Therefore, identifying novel molecular targets and developing safe and effective targeted therapeutics are still urgent for PC prevention and therapy.

**Fig. 1:**
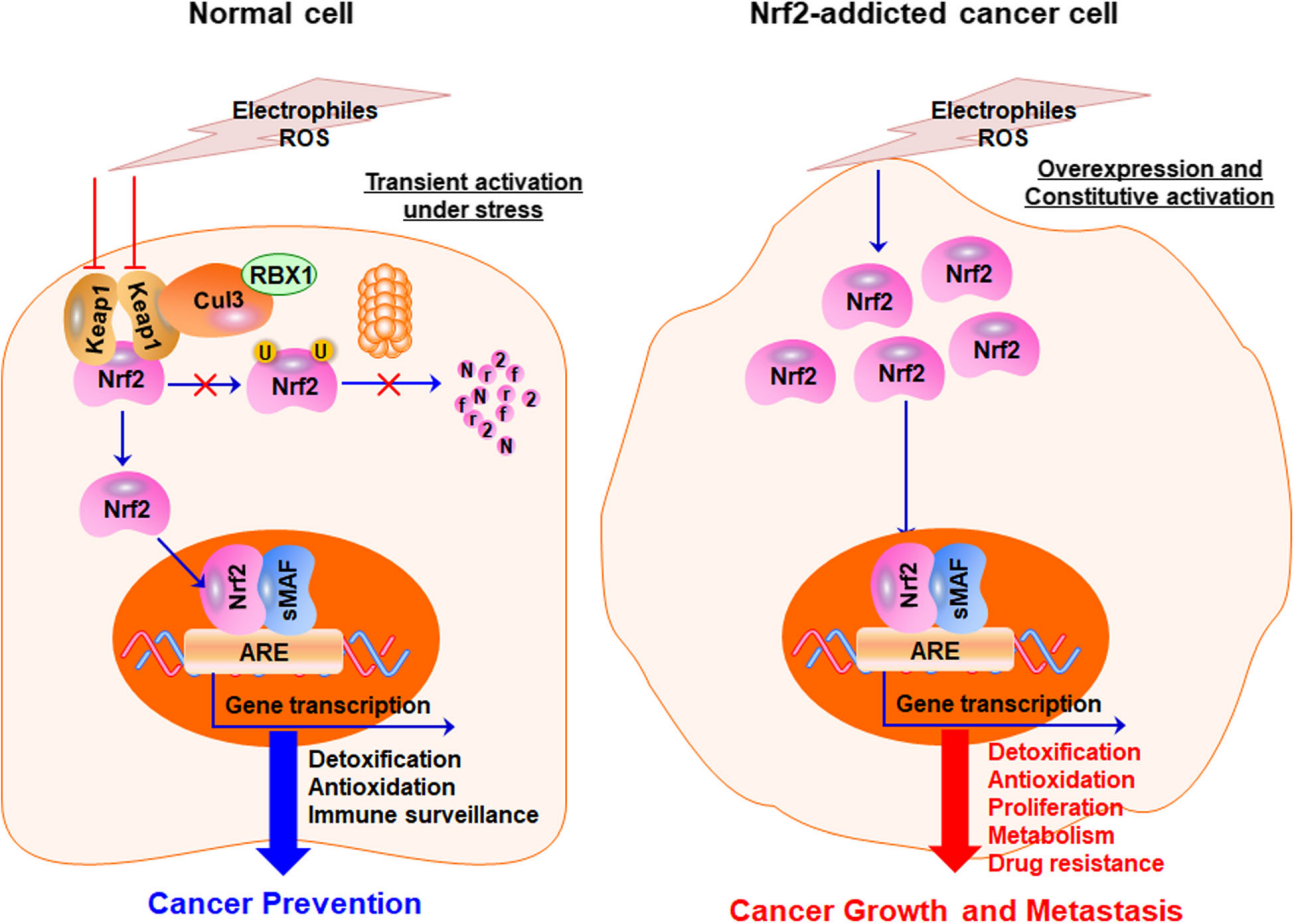
The dual roles of Keap1-Nrf2 signaling pathway in pancreatic cancer. In normal cells, Nrf2 is temporarily activated when exposed to electrophiles and ROS and activates the transcription of genes that increase the capabilities of detoxification, antioxidant, and immune surveillance, preventing chemical-induced carcinogenesis. In Nrf2-addicted cancer cells, Keap1 is deleted or expressed at a very low level. Nrf2 is overexpressed and constitutively activated and promotes cancer growth and metastasis by regulating its downstream target genes

The abnormal expression and activation of the transcription factor nuclear factor-erythroid 2-related factor 2 (Nrf2) and its major negative regulator Kelch-like ECH-associated protein 1 (Keap1) have been observed at different stages of PC and correlated with its initiation, progression, metastasis, and chemoresistance (as shown in Fig. 1) [9, 10]. During the early stage of pancreatic carcinogenesis, Nrf2 exerts a tumor-suppressive role by binding to antioxidant response elements (AREs) and activating its downstream target genes that regulate the cellular antioxidant/detoxification response and immune surveillance [11–13]. However, in the PC progression and metastasis phases, Keap1 mutation and silencing are frequently observed and cause the aberrant stabilization of Nrf2 [9]. Consequently, Nrf2 is constitutively activated and promotes PC growth, metastasis, and chemoresistance by regulating the downstream genes that are involved in proliferation, cell cycle progression, apoptosis, ferroptosis, senescence, autophagy, stem cell self-renewal, angiogenesis, metastasis, drug resistance, and metabolic reprogramming [9, 10, 14, 15]. Recent studies have also shown the preventive efficacy of Nrf2 activators in pancreatic tumorigenesis and the suppressive effects of Nrf2 inhibitors on PC growth and metastasis [16–19]. Because of the opposite roles of Nrf2 at the early and progression stages of PC, a better understanding of the Keap1-Nrf2 pathway and its role in PC may provide a promising strategy for developing novel preventive and therapeutic agents for this dreadful disease.

In the present review, we comprehensively discuss the new findings related to the regulation of the Keap1-Nrf2 pathway, its target genes, and its dual roles in PC initiation, progression, metastasis, and drug resistance. We also review various activators and inhibitors of Nrf2 as well as the current strategies for identifying new Nrf2-targeted agents for PC prevention and therapy.

## Structures of Keap1 and Nrf2 proteins

**Fig. 2:**
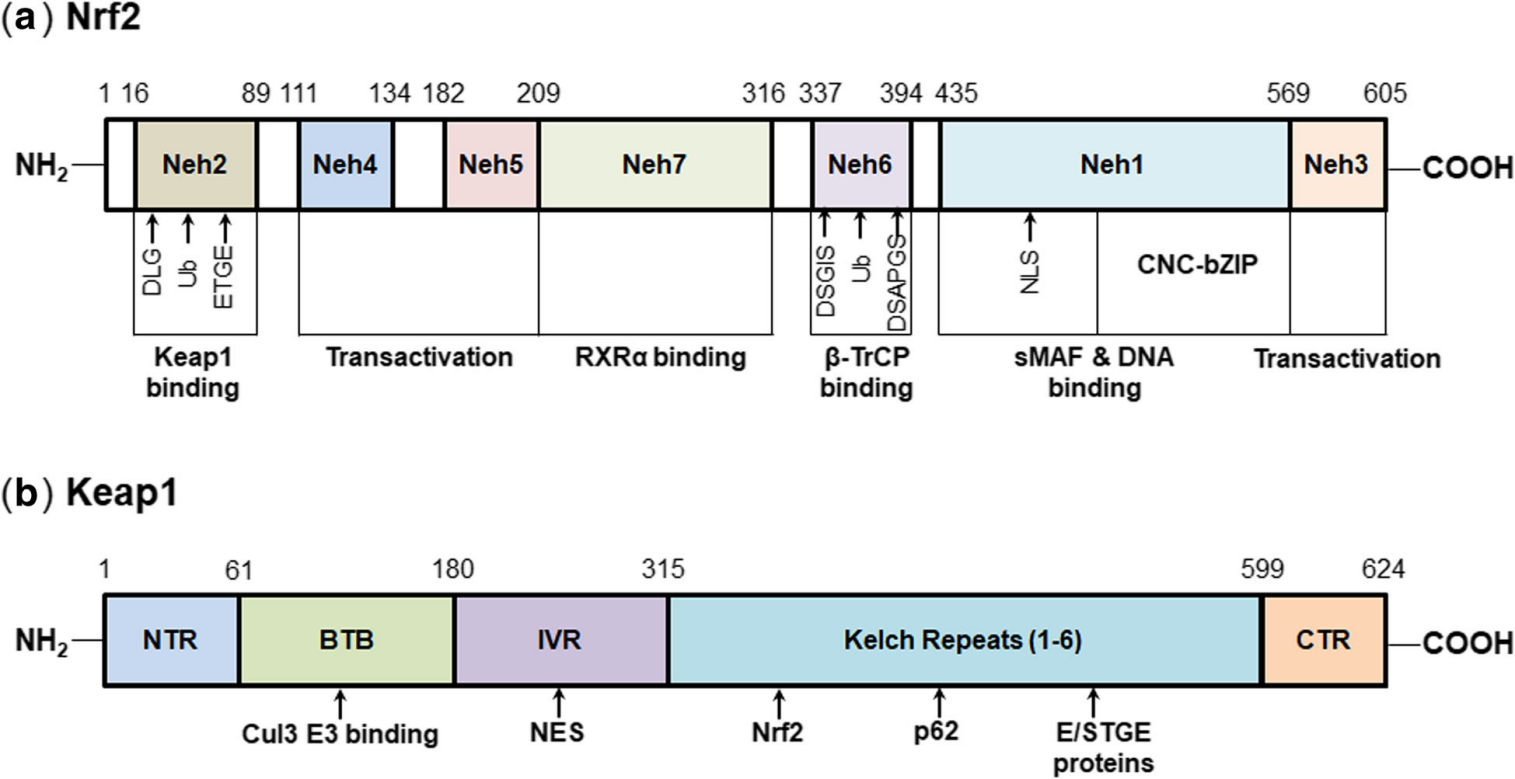
Schematic structures of Keap1 and Nrf2. **a** Nrf2 comprises seven Nrf2-ECH homology (Neh) domains, Neh1-Neh7. Among these domains, Neh2 and Neh6 are important for binding with the negative regulators Keap1 and β-TrCP, respectively, consequently causing Nrf2 ubiquitination and degradation. Neh1 contains a cap ‘n’ collar (CNC) basic-region leucine zipper (bZIP) domain that is important for interacting with small MAF (sMAF) proteins and DNA. Neh1 also holds a nuclear localization signal (NLS) which is required for the nuclear translocation of Nrf2. Neh3, Neh4, and Neh5 domains are necessary for transactivation. Neh7 is important for binding with an Nrf2 repressor, the retinoic X receptor α (RXRα). **b** Keap1 comprises an N-terminal region (NTR), a broad complex, Tramtrack and Bric-à-Brac (BTB) domain, an intervening region (IVR), six Kelch repeats, and a C-terminal region (CTR). Among these domains, BTB domain is responsible for the homodimerization of Keap1 and the binding with Cullin3 (Cul3) E3 ligase. BTB also harbors cysteine residues, which are reactive to electrophiles and reactive oxygen species (ROS). Kelch repeats contain binding sites that are important for interacting with Nrf2, p62, and other E/STGE proteins. IVR contains a nuclear export signal (NES), which regulates the cytoplasmic localization of Keap1

The transcription factor Nrf2 contains 605 amino acids distributed into seven highly conserved Nrf2-ECH homology (Neh) domains, Neh1-Neh7, as depicted in Fig. 2a. Among these domains, Neh1 has a cap ‘n’ collar (CNC) basic-region leucine zipper (bZIP) domain, which is critical for binding to DNA and forming heterodimers with small MAF (sMAF) proteins [20]. In addition, Neh1 contains a nuclear localization signal (NLS) that allows the nuclear translocation of Nrf2 [21]. The Neh2 domain is located in the N-terminal region and contains lysine residues. Neh2 is mainly responsible for the binding with Keap1 homodimers as well as the subsequent ubiquitination and proteasomal degradation of Nrf2 [22]. In addition, Neh6, rich in serine residues, is another negative regulatory domain by binding to β-transducin repeat-containing protein (β-TrCP) and causing Nrf2 ubiquitination and degradation [23]. Conversely, the Neh3 domain endows the Nrf2 protein better stability [24]. In addition, Neh3, Neh4, and Neh5 are transactivation domains that interact with other coactivators [24, 25]. Neh7 is a newly defined domain that is necessary for the binding of Nrf2 to retinoic X receptor α (RXRα), resulting in the inhibition of the Nrf2-ARE signaling pathway [26].

Keap1, the key Nrf2 negative regulator, has 624 amino acids that are distributed into five domains (as shown in Fig. 2b), including an N-terminal region (NTR), a broad complex, Tramtrack and Bric-à-Brac (BTB) domain, a cysteine-rich intervening region (IVR), six Kelch repeats, and a C-terminal region (CTR) [27]. Among these domains, the BTB domain is critical for the homodimerization of Keap1 and the binding with Cullin3 (Cul3) E3 ligase [28]. The Kelch repeats are required for the binding of Keap1 with the Neh2 domain of Nrf2 as well as p62 and other E/STGE-containing proteins [29, 30]. IVR is located between BTB and Kelch repeats and has a nuclear export signal (NES) that regulates the cytoplasmic localization of Keap1 [31]. In addition, Keap1 is highly rich in cysteine residues that act as sensors for electrophiles and reactive oxygen species (ROS), which is important for protecting Nrf2 from proteasomal degradation [32].

## Regulation of Keap1-Nrf2 signaling pathway in pancreatic cancer

### Keap1-Nrf2 signaling pathway

**Fig. 3:**
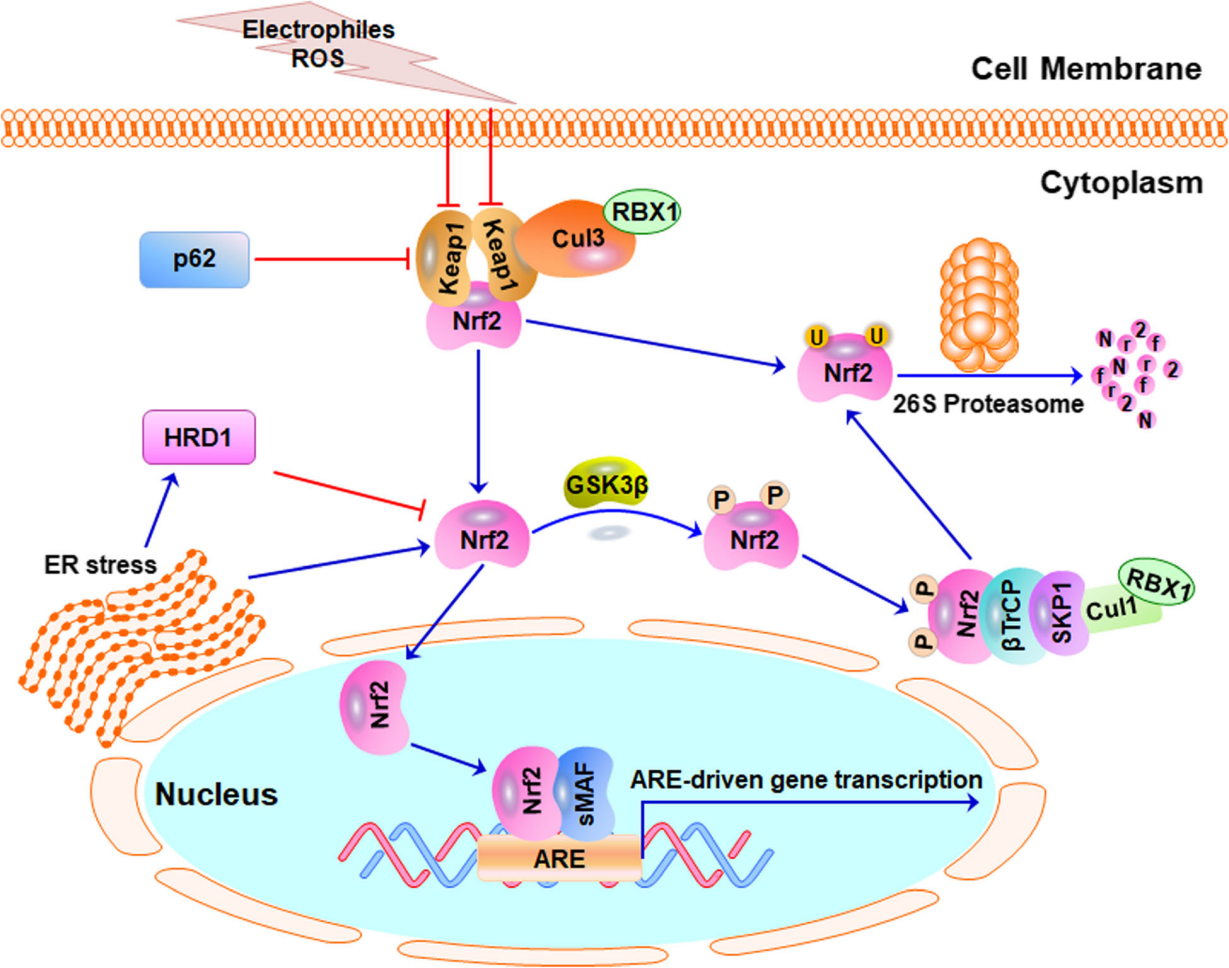
The Keap1-Nrf2 signaling pathway. Under normal physiological conditions, the Nrf2 protein level is tightly controlled by Keap1. Keap1 dimerizes through the N-terminal BTB domain and forms E3 ubiquitin ligase complexes with Cullin3 (Cul3) and Ring box protein-1 (RBX1), then promoting Nrf2 ubiquitination and degradation. Nrf2 is also negatively regulated by the E3 ubiquitin ligase complexes, the β-TrCP-SKP1-Cullin1 (Cul1)-RBX1 and HRD1. When cells are exposed to electrophiles or ROS or under endoplasmic reticulum (ER) stress, the Nrf2 protein level is increased. Nrf2 then translocates into the nucleus, forms heterodimers with sMAF proteins, binds to the antioxidant response elements (AREs), and then activates the transcription of ARE-driven genes. p62 also interacts with the Nrf2-binding site on Keap1 and releases Nrf2 from Keap1-mediated protein degradation

The Keap1-Nrf2 signaling pathway has been extensively reviewed recently [33–35]. Here, we present a brief overview of the Keap1-Nrf2 signaling pathway and its key regulators which are depicted in Fig. 3. Under normal physiological conditions, Nrf2 activity and stability are tightly controlled by Keap1 [33]. Keap1 homodimer directly interacts with Cullin3 (Cul3) and forms the Keap1-Cul3-RBX1 (Ring box protein-1) E3 ligase complex, which targets Nrf2 and induces its polyubiquitination and protein degradation by the 26S proteasome [36]. Under oxidative stress, electrophiles and ROS react with the cysteine residues, especially cysteine 151 in Keap1, which leads to the alteration of Keap1 conformation and its inactivation. Consequently, Nrf2 is released from Keap1-Cul3-RBX1 complex and translocates into the nucleus. The nuclear Nrf2 then forms heterodimers with sMAF proteins and binds to the AREs, activating the transcription of ARE-driven genes [33].

Nrf2 is also negatively regulated by the E3 ubiquitin ligase complex β-TrCP in a glycogen synthase kinase 3β (GSK-3β)-dependent but Keap1-independent manner (Fig. 3) [37]. Nrf2 is phosphorylated by GSK3β at Ser342 and Ser347 residues in the Neh6 domain, which is required for the binding of Nrf2 to β-TrCP as well as the subsequent ubiquitination and degradation [23]. In addition, the E3 ubiquitin ligase HRD1 also compromises Nrf2 activation under endoplasmic reticulum (ER) stress and ROS, independent of Keap1 and β-TrCP [38]. The activation of the ER stress pathway upregulates X-box-binding protein 1 (XBP1)-mediated expression of HRD1, which then directly interacts with Nrf2 and promotes its ubiquitination and proteasomal degradation (Fig. 3) [38]. Moreover, the autophagy substrate p62 directly interacts with the Nrf2-binding site on Keap1, competitively inhibits the binding of Nrf2 to Keap1, and protects Nrf2 from Keap1-Cul3-RBX1-mediated protein degradation [30].

### Direct regulation of Keap1 in pancreatic cancer

Several Keap1 regulators have recently been found to modulate the expression of Keap1 and Keap1-mediated Nrf2 degradation, consequently affecting PC growth and metastasis (as summarized in Table 1). The epigenetic regulator UHRF1 (ubiquitin-like containing PHD and RING finger domains 1) is overexpressed in PC and correlated to tumor growth [39]. Recent studies have shown that UHRF1 suppresses Keap1 expression by inducing *KEAP1* promoter methylation, which causes Nrf2 activation and promotes PC cell proliferation and cell cycle progression [39]. Another epigenetic repressor, MBD1 (methyl-CpG binding domain protein 1) is also highly expressed in PC and negatively regulates the expression of Keap1 by enhancing *KEAP1* promoter methylation, in which c-Myc plays a critical role [40].

**Table 1:** Regulators of Keap1-Nrf2 pathway and their biological effects in pancreatic cancer

Regulators	Keap1/Nrf2	Biological consequences	Refs
Cullin3	Keap1	Forms the Keap1-Cul3-RBX1 E3 ligase complex and induces Nrf2 polyubiquitination and proteasomal degradation	[36]
UHRF1	Keap1	Down-regulates Keap1 expression by enhancing *KEAP1* promoter methylation	[39]
MBD1	Keap1	Down-regulates Keap1 expression by enhancing *KEAP1* promoter methylation	[40]
p62	Keap1	Directly binds to Keap1, competitively inhibits the binding of Nrf2 to Keap1, and protects Nrf2 from protein degradation	[30]
aPKCι	Keap1	Directly binds to Keap1, competitively inhibits the binding of Nrf2 to Keap1, and protects Nrf2 from protein degradation	[41]
PALB2	Keap1	Directly binds to Keap1, competitively inhibits the binding of Nrf2 to Keap1, and protects Nrf2 from protein degradation	[42]
KRAL	Keap1	Directly interacts with miR-141 and increases the expression of Keap1	[43]
β-TrCP	Nrf2	Directly binds to Nrf2 and promotes its ubiquitination and proteasomal degradation	[23]
HRD1	Nrf2	Directly interacts with Nrf2 and promotes its ubiquitination and proteasomal degradation	[38]
GSK-3β	Nrf2	Phosphorylates Nrf2 at Ser342 and Ser347 in Neh6 domain	[23]
ALDOA	Nrf2	Increases the expression of Nrf2	[44]
Kras/ERKaxis	Nrf2	Increases the expression of Nrf2	[45–47]
GRP78/UPR	Nrf2	Increases the activity of Nrf2	[48]
STAT3	Nrf2	Increases the expression of Nrf2	[14]
APE1/Ref-1	Nrf2	Directly interacts with Nrf2 and inhibits its activity	[49]
dCK	Nrf2	Negatively regulates the NRF2 transcriptional activity	[50]
NRAL	Nrf2	Directly binds to miR-340-5p, inhibits miR-340-5p-mediated repressing activity of the Nrf2–3’UTR, and increases Nrf2 expression	[51]

In addition to p62, two new Keap1-binding proteins, aPKCι (atypical protein kinase Cι) and PALB2 (partner and localizer of BRCA2) have been reported to inhibit ROS and promote tumor growth and drug resistance by inducing Nrf2 accumulation, nuclear translocation, and activation (Table 1) [41, 42]. Mechanistically, aPKCι and PALB2 share with Nrf2 a highly conserved Keap1-binding motif and compete with Nrf2 for Keap1 binding, protecting Nrf2 from Keap1-mediated protein degradation [41, 42]. Moreover, microRNAs (miRNAs) and long non-coding RNAs (lncRNAs) have also been found to regulate the expression of Keap1 in PC [43, 52]. A lncRNA, KRAL (Keap1 regulation-associated lncRNA) directly interacts with miR-141as a competing endogenous RNA (ceRNA) and increases the expression of Keap1, leading to the inactivation of Nrf2 and the enhancement of chemosensitization to cancer cells [43].

### Direct regulation of Nrf2 in pancreatic cancer

Except for Keap1-mediated Nrf2 degradation, several regulators have been reported to modulate the expression and transcriptional activity of Nrf2 in PC in a Keap1-independent manner (as summarized in Table 1). The oncogene aldolase A (ALDOA) promotes PC cell proliferation and invasion by increasing the expression of its downstream targets, including Nrf2 [44]. The oncogene Kras also increases the expression of Nrf2, in which ERK (extracellular signal-regulated kinase) and PIN1 (Peptidyl-prolyl cis/trans isomerase NIMA-interacting 1) play a critical role [45–47]. The Kras/ERK/Nrf2 signaling pathway has also been found to promote PC cell growth and cause drug resistance [47]. Also, the glucose regulatory protein 78 (GRP78)-mediated unfolded protein response (UPR) increases the Nrf2 activity and the resistance of PC cells to gemcitabine [48]. The transcription factor STAT3 (signal transducer and activator of transcription 3) has also been shown to upregulate the expression of Nrf2 and induce EMT (epithelial-to-mesenchymal transition) in PC [14].

Several negative regulators of Nrf2 have been found and correlated to PC growth, metastasis, and drug resistance (Table 1). Apurinic/apyrimidinic endonuclease/redox factor-1 (APE1/Ref-1) directly interacts with Nrf2 and inhibits its activity in PC cells [49]. Ref-1 also associates with the *HMOX-1* (heme oxygenase-1) promoter via the Nrf2-binding site, competitively inhibits the Nrf2-*HMOX-1* promoter binding, and down-regulates the expression of HMOX-1 [49]. Decreased deoxycytidine kinase (dCK) has been shown to inhibit PC cell growth and metastasis and sensitize PC cells to gemcitabine treatment by down-regulating the Nrf2 transcriptional activity and decreasing the expression of ARE-driven antioxidant genes [50]. LncRNAs and miRNAs are also involved in the regulation of Nrf2 expression in PC [52]. A lncRNA, termed NRAL (Nrf2 regulation-associated lncRNA) directly binds to miR-340-5p and inhibits the miR-340-5p-mediated repressing activity of the Nrf2–3’UTR, therefore increasing the expression of Nrf2 and inducing drug resistance in PC cells [51].

## The dual roles of Keap1-Nrf2 signaling pathway in pancreatic cancer initiation and progression

The Keap1-Nrf2 signaling pathway has been shown to affect PC initiation and progression in a dual manner, which mainly depends on the stages of this disease and the statuses of Keap1 and Nrf2. In the early phase of pancreatic tumorigenesis, Nrf2 activation prevents carcinogen-induced carcinogenesis by activating the transcription of genes (as summarized in Table 2) that regulate detoxification, antioxidation, and immune surveillance whereas Keap1 inhibits Nrf2 stability and activity and promotes pancreatic carcinogenesis [12, 13, 74, 80, 81]. However, at the stage of cancer progression, the loss-of-function mutations in *Keap1* and the activating mutations in *Nrf2* often happen and lead to the disruption of Keap1-Nrf2 binding and the stabilization and constitutive activation of Nrf2, herein increasing the expression of genes (as summarized in Table 2) that are important for cancer cell proliferation, stem cell self-renewal, cell cycle arrest, apoptosis, ferroptosis, senescence, autophagy, angiogenesis, metastasis, drug resistance, metabolic reprogramming, genome stability and proteotoxic stress [9, 12, 18, 53–73, 75–79, 82]. Of note, somatic mutations in *Keap1* (e.g. G333C, G350S, L413R, D236H) and the methylation of the *Keap1* promoter are frequently observed in lung cancer whereas *Nrf2* mutations (e.g. W24C, E82D, D77V) happen more often in esophageal cancer, which have been reviewed recently [83]. However, there is few study on the accurate detection of mutations in Keap1 and Nrf2 in pancreatic cancer, and the detailed investigations on these mutations are urgently needed.

**Table 2:** Downstream target genes of Nrf2 in pancreatic cancer

Functions	Downstream target genes	Refs
Antioxidant	Upregulated: *NQO1, HO-1, SOD1, GCLC, HMOX1*	[12]
Proliferation	Upregulated: *NOTCH1, NPNT, BMPR1A, IGF1, ITGB2, PDGFC, VEGFC, JAG1*	[53, 54]
Serine and glycine biosynthesis	Upregulated: *ATF4, PHGDH, PSAT1, PSPH, SHMT1, SHMT2*	[55, 56]
Stem cell self-renewal	Upregulated: *NOTCH1, SIRT1, OCT4, NANOG, SOX2, BMI-1, BCL-2, TERT*	[57, 58]
Cell cycle arrest	Upregulated: *CDKN2B, CDKN1A, MDM2*	[54, 59, 60]
Apoptosis	Upregulated: *BCL-2, BCL-xL, HIPK2* Downregulated: *NAF-1*	[61]
Ferroptosis	Upregulated: *MT-1G, FTL1, TH1, SLC11A3, AKR1C1, GPX4*	[62, 63]
Senescence	Upregulated: *MDM2, NOTCH1*	[57, 64]
Autophagy	Upregulated: *SQSTM1/p62, CALCOCO, ULK1, ATG5, GABARAPL1*	[65]
Angiogenesis	Upregulated: *NQO1, HMOX1, G6PD, PGK, TALDO, SLC7A11, PDGFC, FGF2*	[66, 67]
Metastasis	Upregulated: *NOTCH1, MMP2, MMP9, SPP1, GLO1, CDH2, FN1, TWIST2, SNAI1, SLUG*	[14, 53, 68, 69]
Drug resistance	Upregulated: *MRP1, MRP2, MRP3, MRP4, MRP5, ABCG2*	[12, 18, 70]
Metabolic reprogramming	Upregulated: *G6PD, PGD, TKT, TALDO1, ME1, IDH1, PPAT, MTHFD2, GLS* Downregulated: *ACL, ACC1, FASN, SCD1, FADS1, FADS2, ELOVL2, ELOVL6*	[71–73]
Immune surveillance	Upregulated: *ATF3, IL-17D* Downregulated: *IL-6*	[13, 74]
Genome stability	Upregulated: *OGG1,53BP1*	[75, 76]
Proteotoxic stress	Upregulated: *HMOX1, HSP70, SQSTM1/p62, ATF3, PSMA1, PSMA4, PSMA5, PSMB3, PSMB6, PSMC1, PSMC3, PSMD4, PSMD14, POMP*	[77–79]

Nrf2 also promotes mRNA translation and supports efficient protein syntheses, which are partially responsible for Nrf2-regulated redox homeostasis, initiation, and maintenance of PC [10]. More importantly, the high nuclear expression of Nrf2 predicts a worse survival of PC patients, and Nrf2 may be a promising prognostic factor in PC [84]. Recently, Nrf2 has also been found to be elevated in early precursor lesions in the pancreas and contributes to pancreatic carcinogenesis [85–87]. These controversial results can at least partially be explained by recent analyses of the Nrf2-responsive genes in PC cells, which have shown that Nrf2 regulates the expression of both oncogenes and tumor suppressor genes [9, 88]. In this section, we present a comprehensive overview of the dual roles of the Keap1-Nrf2 pathway in PC.

### The tumor suppressive role of Keap1-Nrf2 pathway in pancreatic cancer

Recent studies have provided new evidence showing the preventive effects of Nrf2 on pancreatic carcinogenesis and pancreatic tumor growth and metastasis. Kim et al have recently shown that Nrf2 activation by oxidative stress protects pancreatic beta cells from damage and apoptotic cell death, which is important for preventing pancreatic carcinogenesis induced by oxidants and carcinogens [89]. Nrf2 activation by a natural product has also been found to inhibit PC cell growth and induce apoptosis by upregulating HO-1, although the direct association of Nrf2 activation and the anticancer efficacy still needs further investigation [90]. Satoh et al have demonstrated a critical role of Nrf2 in preventing lung metastasis using an *Nrf2*-null (*Nrf2*^−/−^) and *Keap1*-knockdown (*Keap1*^f/f^) mouse model [91]. It has been reported that Nrf2 is highly expressed in *Keap1*^f/f^ mice, decreases the ROS in the immunosuppressive myeloid-derived suppressor cells (MDSCs), and prevents cancer cell metastasis to the lung after implantation of a lung carcinoma cell line 3LL [91]. However, the Nrf2-deficient *Keap1*^f/f^ mice showed a significantly higher lung metastatic rate after they lost the ability to maintain the redox balance in the immune and hematopoietic systems [91].

### The carcinogenic role of Keap1-Nrf2 pathway in pancreatic cancer

Despite the protective effects of Nrf2 on oxidative stress-induced carcinogenesis, a large number of studies have demonstrated a carcinogenic role of the Keap1-Nrf2 pathway in PC. Previous studies have shown that the Kras oncogene induces Nrf2 expression via the Kras/ERK/NRF2 axis [47, 92]. Kras-mediated Nrf2 expression and activation causes low intracellular ROS levels and promotes pancreatic tumorigenesis and metastasis while Nrf2 inhibition blocks Kras-induced cell proliferation, tumorigenesis, and metastasis [46, 85, 87, 93]. Namada et al have recently examined the role of Nrf2 in pancreatic carcinogenesis using a mouse model carrying pancreas-specific Kras and p53 mutations, named KPC mouse model [85]. It has been found that *Nrf2* deletion in the KPC mice causes a decrease in the formation of precancerous lesions and slows down the development of invasive pancreatic cancer. Furthermore, two cell lines from *Nrf2* deleted KPC tumors (KPCN) have been established and lack the expression of Nrf2 and its downstream target genes, including NQO1. However, the KPCN-derived cell lines still maintain similar features as the cell lines from KPC tumors, except for their increased sensitivity to oxidative stress and gemcitabine [85].

Interestingly, the simultaneous activation of Kras and Nrf2 by Kras mutation and Keap1 deletion, respectively, does not promote PC development as expected but causes pancreatic atrophy [94]. Namada et al have also generated a pancreatic cancer mouse model harboring pancreas-specific *K-ras* mutation and *Keap1* deletion (KC::Keap1) and a KPC mouse model carrying *Keap1* deletion (KPC::Keap1) [94]. Both KC::Keap1 and KPC::Keap1 mice contain constitutively activated Nrf2 due to *Keap1* deletion, which does not promote the development of pancreatic cancer but causes the progressive atrophy of pancreatic parenchyma. Consequently, these *Keap1* deletion mice become weak and even start to die around 40 days after birth [94]. Further studies have shown that *Nrf2* deletion can rescue the phenotypic changes in KC::Keap1 mice, which confirms the role of Nrf2 in pancreatic atrophy. Kras also activates Nrf2 by driving the expression of UHRF1, which preserves *Keap1* promoter methylation and inhibits Keap1 expression [39]. Deletion of UHRF1 inhibits PC cell growth and induces cell cycle arrest at G2/M phase and apoptosis by reducing the expression of Nrf2 and increasing ROS level. However, concomitant deletion of Keap1 and UHRF1 rescues cells from G2/M phase arrest by restoring Nrf2 expression [39].

Nrf2 exerts its carcinogenic role not only by activating the transcription of its target genes but also by promoting mRNA translation [10, 34, 95]. Kha et al have recently discovered that Nrf2 activation protects premalignant pancreatic ductal epithelial (PDE) cells from apoptosis and accelerates the formation and growth of pancreatic tumors by inducing the expression of a splicing variant of ATF3 (activating transcription factor 3), termed ΔZip2 [96]. Nrf2 also attenuates TGF-β1 (transforming growth factor-β1)-mediated growth inhibition of PDE cells by decreasing the expression levels of p21, phosphor-p38 and phosphor-Smad3 [86]. Moreover, Nrf2 inhibits PC cell apoptosis by upregulating the expression of anti-apoptotic proteins (e.g., Bcl2) and other cytoprotective proteins and enzymes [97].

Pancreatic stellate cells (PSC)-secreted SDF-1α (stromal-derived factor-1α) and IL-6 (interleukin-6) activates Nrf2, which further induces metabolic reprogramming and ROS detoxification and promotes PC cell proliferation [98]. Further studies have shown that PSC-secreted IL-6 also promotes EMT in PC cells by activating the JAK (Janus kinase)/STAT3/Nrf2 pathway. It has been observed that Nrf2 activation upregulates the expression of N-cadherin, fibronectin, Twist2, Snail, and Slug, which contribute to the increased EMT phenotypes [14]. Nrf2 has also been reported to induce EMT by regulating the cancer cells and macrophages interaction [99]. PC cells elevate the intracellular ROS level in macrophages via lactate secretion, which further activates Nrf2, induces macrophage M2 phenotype transformation, and increases VEGF (vascular endothelial growth factor) expression. The cancer cell-educated macrophages then induce Nrf2 activation in PC cells via VEGF secretion and promote EMT [99].

Pancreatitis-induced autophagy substrate p62 has been found to promote PC progression by activating the Keap1/Nrf2/MDM2 (murine double minute 2) signaling pathway [60, 100]. Mechanistically, p62 accumulation stabilizes Nrf2 protein and increases Nrf2-mediated MDM2 expression, which further accelerates the progression of pancreatic intraepithelial neoplasia (PanIN) to PC through both p53-dependent and p53-independent mechanisms [60]. These results also support that directly inhibiting MDM2 may be effective in preventing PanIN to PC progression, regardless of the p53 status [101, 102]. In addition to the regulation by the autophagy adaptor p62, Nrf2 has been reported to promote autophagy by targeting miR-129-3p in PC cells under the treatment of histone deacetylase inhibitors (HDACis) [103]. Further studies have indicated that mammalian target of rapamycin (mTOR) is a target of miR-129-3p and the Nrf2-miR-129-3p-mTOR axis is mainly responsible for HDACis-induced autophagy in PC cells [103]. However, Nrf2 activation has also shown a negative interaction with autophagy under ROS stress [104]. ROS stimulation induces both Nrf2 activation and autophagy in PC cells and inhibiting either Nrf2 or autophagy will lead to the enhancement of another one [104]. Interestingly, the combined inhibition of Nrf2 pathway and autophagy also increases PC cell apoptosis by chemotherapy, which may provide a new treatment approach for PC patients [105].

The Keap1-Nrf2 pathway is also involved in chemoresistance in PC by regulating the expression of drug resistance-associated genes and cytoprotective antioxidant genes (Table 2) [15, 106]. Furthermore, ALDH1A1 (aldehyde dehydrogenase 1 family, member A1) and ALDH3A1 (aldehyde dehydrogenase 3 family, member A1) are up-regulated by Nrf2 and may also contribute to drug resistance in PC [107]. LncRNAs, KRAL and NRAL have been reported to exert an opposite role in mediating the drug resistance by regulating the miR-141/Keap1 axis and the miR-340-5p/Nrf2 axis, respectively [43, 51]. Chemotherapies, e.g. gemcitabine have also been found to increase Nrf2 expression, while pretreatment with Nrf2 inhibitors enhances the sensitivity of PC cells to chemotherapy [108].

## Targeting Keap1-Nrf2 signaling pathway for pancreatic cancer prevention and therapy

**Fig. 4:**
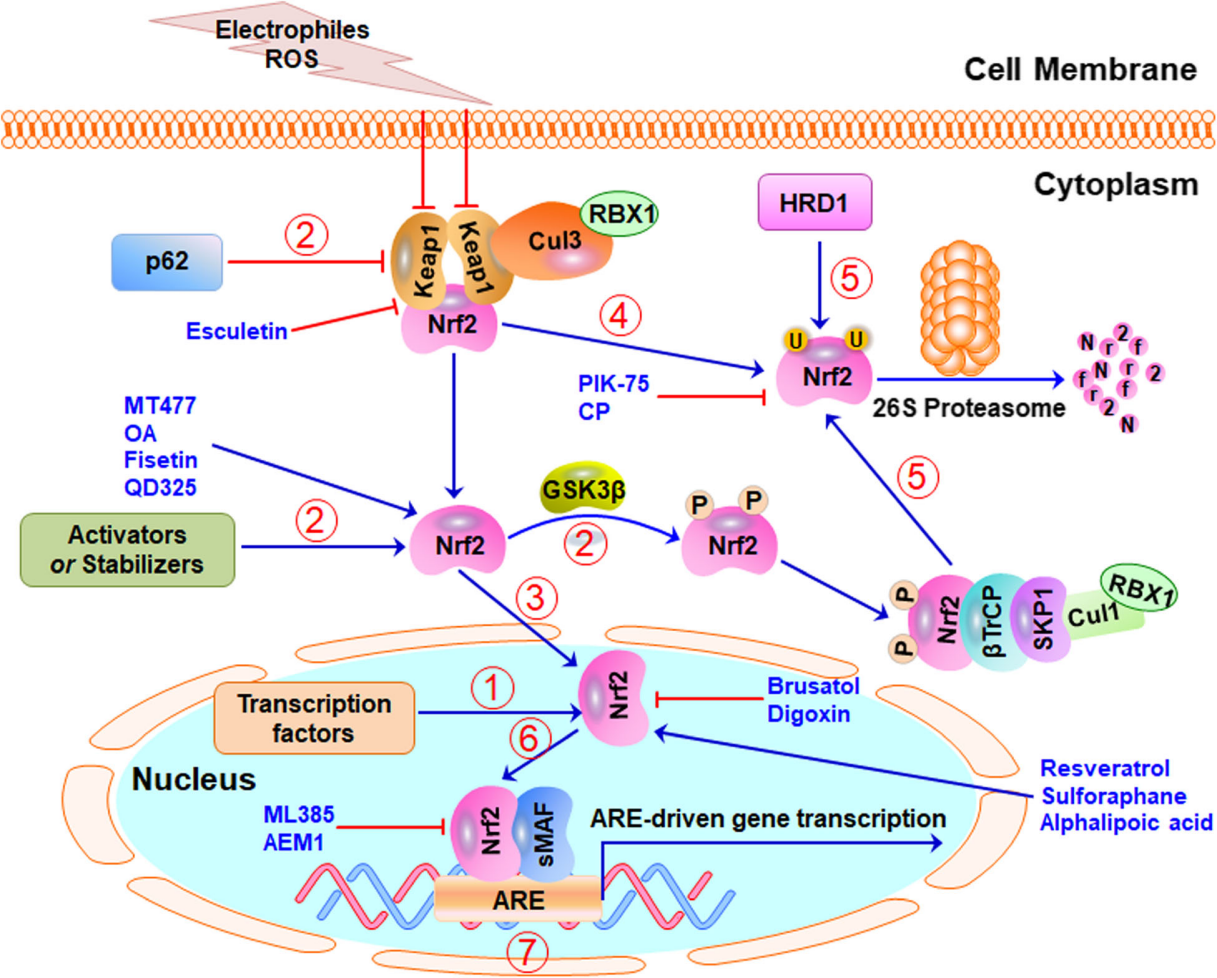
Targeting Keap1-Nrf2 signaling pathway for pancreatic cancer prevention and therapy. Several strategies have been proposed to target the Nrf2 signaling pathway in human pancreatic cancer: (1) modulating Nrf2 expression at the transcriptional level, (2) modulating the Nrf2 activity by targeting its upstream activators and stabilizers, (3) affecting the nuclear translocation of Nrf2, (4) targeting the Keap1-Nrf2 binding for modulating Nrf2 protein stability, (5) targeting the β-TrCP-Nrf2 binding or the HRD1-Nrf2 binding and modulating Nrf2 ubiquitination and degradation, (6) modulating the binding of Nrf2 with its co-activators in the nucleus, and (7) modulating the binding of Nrf2 with its downstream target genes. Many small-molecule Nrf2 activators and inhibitors have been discovered and shown efficacy in pancreatic cancer cells in vitro and in vivo

The Keap1-Nrf2 pathway has been considered as a potential target for PC prevention and therapy. Several Nrf2 activators and inhibitors have been identified and shown efficacy in PC models in vitro and in vivo (as summarized in Table 3). In this section, we focus on the current activators and inhibitors, their efficacy and mechanisms of action. We also review known targeting strategies and propose new targeting strategies (Fig. 4) that may be used to develop more specific and effective Nrf2-targeted agents for PC prevention and therapy.

**Table 3:** Summary of compounds targeting Keap1-Nrf2 pathway and their mechanisms of action

Compounds	Mechanisms of action	In vitro activity	In vivo activity	Refs
*Nrf2 activators*				
Esculetin	Binds to Keap1, disrupts Keap1-Nrf2 interaction, and activates Nrf2	Inhibits cell growth, arrests cells at G1 phase, and induces cell apoptosis	*NR*	[109]
MT477	Activates Nrf2 signaling pathway	Inhibits cell survival	Suppresses tumor growth in MiaPaca-2 xenograft model	[16]
Oleanolic acid (OA)	Activates ERK/Nrf2 signaling pathway	Suppression of ERK/Nrf2 pathways strengthens OA-induced apoptosis	Suppression of ERK/Nrf2 pathways enhances OA’s efficacy in a xenograft model	[45]
Fisetin	Activates Nrf2 signaling pathway	Inhibits cell growth	*NR*	[110]
QD325	Induces substantial ROS and activates Nrf2 signaling pathway	Inhibits cell growth	Suppresses tumor growth and enhances the efficacy of gemcitabine in MiaPaca-2 xenograft model	[17]
Resveratrol	Increases the expression and activity of Nrf2 and decreases the expression of NAF-1	Inhibits cell growth, induces cell apoptosis, and enhances cell sensitivity to gemcitabine	*NR*	[61]
Sulforaphane	Increases the expression and activity of Nrf2 through activating AMPK	Inhibits cell growth and invasion	Suppresses tumor growth in Panc-1 xenograft model and a transgenic pancreatic cancer mouse	[90]
Alphalipoic acid	Increases the expression and activity of Nrf2	*NR*	Suppresses tumor growth in CFPAC-1 xenograft model	[111]
*Nrf2 inhibitors*				
Brusatol	Inhibits Nrf2 expression and activity and increases ROS accumulation	Enhances growth inhibition and apoptosis caused by gemcitabine	Suppresses tumor growth and enhances the efficacy of gemcitabine in Panc-1 xenograft model	[112]
Digoxin	Decreases *Nrf2* mRNA level through inhibiting PI3K/Akt pathway	Enhances growth inhibition and apoptosis caused by gemcitabine	Sensitizes SW1990/Gem cells-derived xenografts to gemcitabine	[19]
PIK-75	Induces Nrf2 proteasomal degradation	Inhibits cell proliferation and survival and potentiates gemcitabine- induced cytotoxicity	Suppresses tumor growth and enhances the efficacy of gemcitabine in MiaPaca-2 xenograft model	[18]
Clobetasol propionate (CP)	Prevents Nrf2 nuclear accumulation and induces its degradation	Inhibits growth of cancer cells with mutations in Keap1 or both in Keap1 and LKB1 alone or in combination with rapamycin	Suppresses growth of tumors containing mutations in both Keap1 and LKB1 alone or in combination with rapamycin	[113]
ML385	Directly binds to the CNC-bZIP domain of Nrf2 and inhibits its DNA binding activity	Exerts selective cytotoxicity against cancer cells with Keap1 mutations alone or in combination with carboplatin	Suppresses growth of tumors with Keap1 mutations alone or in combination with carboplatin	[114]
AEM1	Inhibits Nrf2 transcriptional activity	Inhibits the growth of cancer cells harboring mutant Keap1 alone or in combination with chemotherapy	Suppresses growth of tumors harboring Keap1 mutations	[115]
Pterostilbene	Inhibits Nrf2 nuclear translocation and activity	Inhibits cell viability	Suppresses tumor growth in AsPC-1 xenograft model	[116]

*NR*, Not reported

### Nrf2 activators

Considering the critical role of Keap1 in regulating Nrf2 stability, targeting the Keap1-Nrf2 binding has been demonstrated as a promising strategy for identifying specific activators of Nrf2 (as shown in Fig. 4). A recent study has discovered a dihydroxy coumarin derivative, termed esculetin that directly binds to Keap1 and inhibits its binding to Nrf2 [109]. The released Nrf2 by esculetin has been observed to reduce ROS level, inhibit cell growth, arrest cells at G1 phase, and induce apoptosis and loss of mitochondrial membrane potential in PC cells in vitro [109]. The activation of caspase cascade and inhibition of NF-κB (nuclear factor-κB) also contribute to the anticancer activity of esculetin. However, the selectivity of esculetin to Keap1 and its in vivo efficacy still need further investigation in clinically relevant models of PC. Because Keap1 is often deleted and mutated in pancreatic cancer cells, which also causes the aberrant activation of Nrf2, disrupting the Keap1-Nrf2 binding for reactivating Nrf2 could not be an effective strategy for PC therapy. Therefore, it is possible that the reactivation of Nrf2 by esculetin may not be mainly responsible for its anticancer activity.

Currently, most of the identified Nrf2 activators, including MT477 [16], oleanolic acid (OA) [45], fisetin [110], QD325 [17], resveratrol [61], sulforaphane (SFN) [90], and alphalipoic acid [111] have been found to increase Nrf2 expression and/or enhance Nrf2 activity (Table 3). Among them, OA and SFN have been demonstrated to activate the upstream regulators Nrf2 pathway, ERK and AMPK (AMP-activated protein kinase), respectively, which are important for the increased expression of Nrf2 by these compounds [45, 90]. However, OA-induced Nrf2 activation has been found to compromise its anticancer activity and the suppression of ERK/Nrf2 pathway enhances OA-induced cancer cell apoptosis in vitro and its inhibitory effects on the growth of xenograft tumors in vivo [45]. Although SFN has been shown to inhibit PC cell growth and invasion in vitro and suppress tumor growth in both Panc-1 xenograft model and a transgenic mouse model in vivo [90], the importance of Nrf2 activation in SFN’s anti-PC activity as well as the detailed molecular mechanisms should be further investigated.

All the other known Nrf2 activators have also been shown to inhibit PC cell survival in vitro and/or suppress the growth of PC xenograft tumors in vivo (Table 3). Among them, both MT477 and QD325 have shown in vivo efficacy in MiaPaca-2 xenograft model [16, 17], whereas alphalipoic acid has been found to inhibit the growth of CFPAC-1 xenograft tumor [111]. However, it is still unknown whether Nrf2 activation is responsible for their anti-PC activities. They may activate Nrf2 by inducing cellular ROS accumulation and/or affecting Nrf2 upstream regulators, which need to be investigated for the detailed molecular mechanisms. More importantly, the mutations in Keap1 and constitutive activation of Nrf2 are frequently observed in various cancers, including PC, the safety and feasibility of using Nrf2 activators for treating PC should be carefully examined.

### Nrf2 inhibitors

The currently identified Nrf2 inhibitors have been reported to suppress Nrf2 expression, induce Nrf2 protein degradation, or inhibit Nrf2 nuclear translocation (as shown in Fig. 4). A natural product termed brusatol has been reported to reduce Nrf2 protein levels without affecting the expression of Keap1 in PC cells [112]. The down-regulation of Nrf2 by brusatol further caused the repression of its downstream targets that are responsible for drug resistance (*MRD1* and *MRP5*) and antioxidant response (*HQO1* and *HO-1*). Because the upregulation of Nrf2 contributes to gemcitabine resistance, brusatol has also been evaluated in combination with gemcitabine. As expected, brusatol significantly enhanced the anticancer activity of gemcitabine in PC cells in vitro and in vivo [112]. However, brusatol has been reported to be a broad inhibitor of protein translation and could not be used as a specific Nrf2 inhibitor. Another natural-product Nrf2 inhibitor, digoxin has been found to down-regulate Nrf2 expression at the transcription level by inhibiting PI3K (Phosphoinositide 3-kinase)/Akt pathway [19]. Consistently, digoxin-induced Nrf2 inhibition also reverses the resistance of gemcitabine-resistant PC cells to gemcitabine in vitro and in vivo [19].

Nrf2 protein stability is mainly mediated by the ubiquitin-proteasome system. There are at least three E3 ubiquitin ligase complexes, including Keap1-Cul3-RBX1 complex, β-TrCP, and HRD1 that control the ubiquitination of Nrf2 [23, 36, 38]. Therefore, inducing Nrf2 ubiquitination and proteasomal degradation may be an effective approach to inhibit Nrf2. PIK-75, a previously reported PI3K/DNA-PK inhibitor has recently been identified to inhibit Nrf2 by inducing its proteasome-mediated protein degradation [18]. Further studies have also shown its inhibitory effects on Nrf2 transcriptional activity. The anticancer efficacy of PIK-75 alone or in combination with gemcitabine has been demonstrated in PC models in vitro and in vivo [18]. Very recently, a drug-repositioning screening of ~ 4000 clinical compounds has been performed and led to the identification of a potent Nrf2 inhibitor, clobetasol propionate (CP) [113]. CP has been shown to prevent Nrf2 nuclear accumulation and promote its protein degradation in a glucocorticoid receptor-, GSK3-, and β-TrCP-dependent manner. More importantly, CP not only inhibits the growth of tumors with Keap1 and/or LKB1 (liver kinase B1) mutations but also enhances the anticancer activity of rapamycin in vitro and in vivo [113]. These results have suggested that CP could be developed as a therapeutic agent for cancers, especially those with Nrf2 activation and Keap1 and LKB1 mutations.

Directly targeting the active domain of Nrf2 has recently been considered as a potential strategy for developing Nrf2 inhibitors. Singh et al have performed a high-throughput screen of a compound library containing ~ 400,000 small molecules and identified a specific Nrf2 inhibitor, termed ML385, which directly binds to the CNC-bZIP domain of Nrf2 and inhibits the DNA binding activity of the MafG-Nrf2 protein complex [114]. Further studies have shown that ML385 exerts selective cytotoxicity against cancer cells with Keap1 mutations and increases the toxicity of carboplatin in vitro and in vivo [114]. However, it is still unclear whether ML385 is a selective Nrf2 inhibitor or a broad inhibitor of various transcription factors. Another high throughput screening has also led to the identification of a novel Nrf2 inhibitor, named AEM1, which can inhibit Nrf2 transcriptional activity and decrease the expression of its target genes [115]. AEM1 alone and in combination with chemotherapy has been shown to inhibit the growth of cancer cells harboring mutant Keap1 and constitutively activated Nrf2 in vitro and in vivo [115]. However, the cellular target of AEM1, as well as its associated molecular mechanism, is still unknown. In addition, the inhibition of nuclear Nrf2 and its transcription activity has been reported to be involved in the anti-PC activity of pterostilbene in vitro and in vivo, but the detailed molecular mechanisms are still not clear yet [116].

As summarized in Fig. 4 and Table 3, most of the known Nrf2 activators and inhibitors have been developed to (1) modulate Nrf2 expression, (2) target Nrf2 upstream regulators, (3) affect the nuclear translocation of Nrf2, or (4) target Keap1-Nrf2 binding. However, there are still several strategies that need to be further examined for developing novel and specific Nrf2-targeted agents, such as (5) affectingNrf2 ubiquitination and degradation by targeting the β-TrCP-Nrf2 binding and HRD1-Nrf2 binding or developing Nrf2-targeted PROTACs (proteolysis targeting chimeras), (6) modulating the binding of Nrf2 with its co-activators in the nucleus, and (7) modulating the binding of Nrf2 with its downstream target genes.

## Conclusions and future direction

During the early stage of pancreatic tumorigenesis, Nrf2 is tightly controlled by Keap1. Nrf2 activation by oxidative stress prevents PC initiation by regulating antioxidant and detoxification response and immune surveillance. In the phases of PC progression and metastasis, Keap1 is mutated or deleted and Nrf2 is constitutively activated. At this stage, the role of Nrf2 is switched from tumor suppression to tumor promotion. Nrf2 can promote pancreatic tumor growth, metastasis, and chemoresistance by activating its downstream target genes and promoting mRNA translation. It has been shown that Nrf2 activators can prevent pancreatic tumorigenesis but may also induce chemoresistance. Conversely, Nrf2 inhibitors can inhibit pancreatic tumor growth and metastasis and sensitize PC cells to chemotherapies, especially gemcitabine. However, the feasibility of the clinical application of Nrf2-targeted agents for PC prevention and therapy still needs further investigation due to the controversial roles of Nrf2 at different stages of PC. Furthermore, the majority of the current Nrf2 activators and inhibitors have been found to have other molecular targets and more specific Nrf2-targeted compounds should be developed. Several unexplored strategies, i.e. targeting the β-TrCP-Nrf2 binding and HRD1-Nrf2 binding or developing Nrf2 PROTACs may be investigated for specific Nrf2-targeted agents for PC prevention and therapy.

## Data Availability

Not applicable.

## Abbreviations

ALDH1A1: Aldehyde dehydrogenase 1 family, member A1
ALDH3A1: Aldehyde dehydrogenase 3 family, member A1
ALDOA: Aldolase A
AMPK: AMP-activated protein kinase
APE1/Ref-1: Apurinic/apyrimidinic endonuclease/redox factor-1
aPKCι: Atypical protein kinase Cι
AREs: Antioxidant response elements
ATF3: Activating transcription factor 3
BTB domain: Broad complex, Tramtrack andBric-à-Brac domain
bZIP: Basic-region leucine zipper
ceRNA: Competing endogenous RNA
CNC: Cap ‘n’ collar
CP: Clobetasol propionate
CTR: C-terminal region
Cul1: Cullin1
Cul3: Cullin3
dCK: Deoxycytidine kinase
EMT: Epithelial-to-mesenchymal transition
ER: Endoplasmic reticulum
ERK: Extracellular signal-regulated kinase
GRP78: Glucose regulatory protein 78
GSK-3β: Glycogen synthase kinase 3β
HDACis: Histone deacetylase inhibitors
HMOX-1: Heme oxygenase-1
IL-6: Interleukin-6
IVR: Intervening region
JAK: Janus kinase
KC::Keap1 mice: Mice harboring pancreas-specific *K-ras* mutation and *Keap1* deletion
Keap1: Kelch-like ECH-associated protein 1
KPC::Keap1 mice: KPC mice bearing *Keap1* deletion
KPCN mice: KPC mice bearing *Nrf2* deletion
KRAL: Keap1 regulation-associated lncRNA
LKB1: Liver kinase B1
LncRNA: Long non-coding RNA
MBD1: Methyl-CpG binding domain protein 1
MDM2: Murine double minute 2
MDSCs: Myeloid-derived suppressor cells
miRNAs: MicroRNAs
mTOR: Mammalian target of rapamycin
Neh: Nrf2-ECH homology
NES: Nuclear export signal
NF-κB: Nuclear factor-κB
NLS: Nuclear localization signal
NRAL: Nrf2 regulation-associated lncRNA
Nrf2: Nuclear factor-erythroid 2-related factor 2
NTR: N-terminal region
OA: Oleanolic acid
PALB2: Partner and localizer of BRCA2
PanIN: Pancreatic intraepithelial neoplasia
PC: Pancreatic cancer
PDE: Pancreatic ductal epithelial
PI3K: Phosphoinositide 3-kinase
PIN1: Peptidyl-prolyl cis/trans isomerase NIMA-interacting 1
PROTACs: Proteolysis targeting chimeras
PSC: Pancreatic stellate cells
RBX1: Ring box protein-1
ROS: Reactive oxygen species
RXRα: Retinoic X receptor α
SDF-1α: Stromal-derived factor-1α
SFN: Sulforaphane
sMAF: Small MAF
STAT3: Signal transducer and activator of transcription 3
TGF-β1: Transforming growth factor-β1
UHRF1: Ubiquitin-like containing PHD and RING finger domains 1
UPR: Unfolded protein response
VEGF: Vascular endothelial growth factor
XBP1: X-box-binding protein 1
β-TrCP: β-transducin repeat-containing protein
